# Identification of Murine Rotavirus Virulence Determinants Using Bidirectional Selective Passaging and a Reverse Genetics System

**DOI:** 10.3390/v18070747

**Published:** 2026-07-06

**Authors:** Saori Fukuda, Masanori Kugita, Yuki Akari, Johannes M. Dijkstra, Yoshiki Kawamura, Shizuko Nagao, Tetsushi Yoshikawa, Takayuki Murata, Satoshi Komoto

**Affiliations:** 1 Department of Virology, Fujita Health University School of Medicine, Toyoake 470-1192, Aichi, Japan; tmurata@fujita-hu.ac.jp; 2 Center for Infectious Disease Research, Research Promotion Headquarters, Fujita Health University, Toyoake 470-1192, Aichi, Japan; tetsushi@fujita-hu.ac.jp; 3 Advanced Medical Research Center for Animal Models of Human Disease, Research Promotion Headquarters, Fujita Health University, Toyoake 470-1192, Aichi, Japan; m-kugi@fujita-hu.ac.jp (M.K.); shizun@fujita-hu.ac.jp (S.N.); 4 Division of One Health, Research Center for GLOBAL and LOCAL Infectious Diseases (RCGLID), Oita University, Yufu 879-5593, Oita, Japan; m24d9001@oita-u.ac.jp (Y.A.); satoshik@oita-u.ac.jp (S.K.); 5 Office of Research Administration, Fujita Health University, Toyoake 470-1192, Aichi, Japan; dijkstra@fujita-hu.ac.jp; 6 Department of Pediatrics, Fujita Health University School of Medicine, Toyoake 470-1192, Aichi, Japan; yoshiki@fujita-hu.ac.jp

**Keywords:** rotavirus, pathogenicity, attenuation, vaccine, reverse genetics

## Abstract

Live-attenuated rotavirus (RV) vaccines are the most effective interventions for preventing RV gastroenteritis (RVGE) in young children. However, the molecular basis of attenuation remains not well understood. Here, we describe a compact but comprehensive strategy to identify RV virulence determinants by combining low-passage bidirectional selection, sequence analysis, and segment-level phenotype testing via a reverse genetics infectious system. Using the virulent murine RV strain EW, virulence was quantified by diarrhea severity/duration and body-weight gain. Serial passaging in cell culture selected an attenuated population, which regained virulence after passaging in suckling mice. Sequence comparison of the virulent and attenuated EW populations revealed only seven amino acid differences. We summarized literature describing attenuation/virulence-associated mutations in various RV group A (RVA) strains and found previous findings identical or similar to four of the seven mutations: NSP4-T45M, VP4-S470L, VP4-T612A, and VP7-T75P. Virulent- and attenuated-type EW variants of VP2, VP4, VP7, and NSP4 were introduced individually, or as NSP4/VP7 or VP4/VP7 pairs, into a simian SA11-L2 backbone using an 11-plasmid reverse genetics system. Phenotyping of rescued viruses consistently linked cell-culture–adapted VP4 to enhanced replication in vitro and reduced virulence in suckling mice. In vivo passaging strongly favored VP4 residue S470 over cell-culture-selected L470. More generally, our findings (i) underscore VP4 and VP7 as key determinants of EW virulence, (ii) provide a practical framework for identifying driver mutations underlying RVA attenuation, and (iii) highlight attenuation-associated substitutions shared across diverse RVAs.

## 1. Introduction

Group A rotavirus (RVA), belonging to the Sedoreoviridae family, is a primary cause of severe gastroenteritis in young children worldwide. Approximately 128,500–215,000 annual deaths among children < 5 years of age are estimated, mainly in developing countries [1,2,3]. The RVA’s double-stranded RNA (dsRNA) genome consists of 11 gene segments encoding six structural (VP1 to VP4, VP6, and VP7) and six nonstructural proteins (NSP1 to NSP6), with the two outer capsid proteins (VP7 and VP4) defining the G and P genotypes, respectively [4]. As of 2026, the Rotavirus Classification Working Group (RCWG) (https://rega.kuleuven.be/cev/viralmetagenomics/virus-classification/rcwg, accessed on 2 July 2026) has recognized at least 42 G and 58 P genotypes; dominant in humans are six G genotypes (G1 to G4, G9, and G12) in combination with three P genotypes (P[4], P[6], and P[8]) [4,5,6,7,8]. Since 2006, two widely used live-attenuated oral RV vaccines, Rotarix^®^ (GlaxoSmithKline Biologicals, Rixensart, Belgium) and RotaTeq^®^ (Merck & Co., Inc., Rahway, NJ, USA), have been licensed in >100 countries worldwide and severe rotavirus disease has declined substantially since the introduction of these vaccines [2,3,9,10,11]. Specifically, among children < 5 years of age, the rate of RVA-associated mortality decreased by 48.2% between 1990 and 2016 [2] and the rate of RVA-associated hospitalization decreased by 59% in 47 countries between 2006 and 2019 [3]. Reductions in mortality and hospitalizations accelerated after vaccine introduction, promoted by the recommendation of the World Health Organization (WHO) to incorporate a rotavirus vaccine in national immunization programs globally [12].

Despite the success of live-attenuated RV vaccines, identifying the molecular basis of their attenuation has been difficult. Obstacles in many previous approaches were that they compared viruses that were not closely related (and therefore differed at many sites) and/or analyzed reassortants generated by co-infection (which limits experimental control) (e.g., [13,14]). If the number of differences is large, this confounds interpretation because phenotypes may reflect combined effects of multiple differences and epistatic interactions, plus some differences may have been acquired incidentally as background variation inherited alongside adaptive substitutions.

In this study, we used the murine RVA strain EW, a prototypic epizootic diarrhea of infant mice (EDIM) rotavirus that serves as a model of species-matched RV pathogenesis [15]. It has already been established that serial passaging of EW in cell culture selects variants with improved replication in vitro that exhibit reduced pathogenicity in neonatal mice [16,17,18].

To our knowledge, this is the first study to combine an authentic murine RV pathogenesis system with a reverse-genetics reassortant approach to gain insight into genome-wide attenuation-associated mutations. Furthermore, compared to other RV attenuation studies, this study is unusually compact and comprehensive. Critical differences compared to many previous studies are the analysis of RV variants after only 10 or fewer passaging events (restricting the number of differences), selection in vitro as well as in vivo to confirm relevance of detected changes, and phenotyping in the natural host utilizing a reverse genetics infectious system. Screening across all 11 viral segments, we found the attenuation/virulence acquisition to be most strongly associated with selection of the outer capsid protein VP4, particularly its serine-versus-leucine difference at position 470.

## 2. Materials and Methods

### 2.1. Cells and Viruses

A baby hamster kidney cell line stably expressing the T7 RNA polymerase (BHK/T7-9) [19] was cultured in Dulbecco’s modified Eagle medium (DMEM; Nacalai, Kyoto, Japan) supplemented with 5% fetal calf serum (FCS; Gibco, Tokyo, Japan) (complete medium) in the presence of 600 ng/mL hygromycin (Invitrogen, Tokyo, Japan). Monkey kidney cell lines, MA104 and CV-1, were cultured in complete medium.

Murine RV strain EW (G16-P[16]-I7-R7-C7-M8-A7-N7-T10-E7-H9) isolated from suckling mice with diarrhea (named EW/v-ori) was kindly obtained from H.B. Greenberg and has been described in detail previously [15]. Recombinant simian RV strain SA11-L2 (G3-P[2]-I2-R2-C5-M5-A5-N5-T5-E2-H5) (rSA11-L2) [20,21] was used as a reverse genetics platform.

### 2.2. Serial Passages in Suckling Mice to Adapt the EW/c-ori or EW/v-ori to Mice

**Figure 1:**
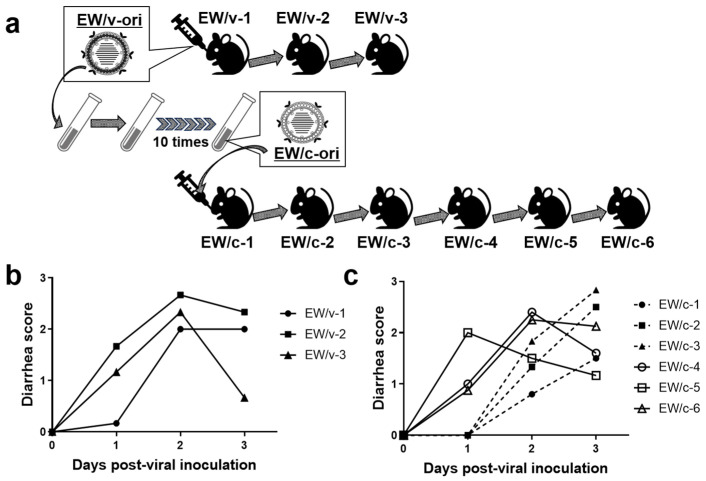
Bidirectional passaging of murine rotavirus EW and in vivo phenotypes. (**a**) Passaging scheme: the virulent EW stock (EW/v-ori) was serially passaged in six-day-old suckling mice (EW/v-1 to EW/v-3; *n* = 6 pups/group). In parallel, EW/v-ori was adapted to MA104 cells by 10 sequential passages to generate EW/c-ori, which was then serially passaged in suckling mice (EW/c-1 to EW/c-6; *n* = 4–8 pups/group). (**b**,**c**) Diarrhea severity over 3 days after oral inoculation with EW/v-ori (**b**) or during serial in vivo passaging of EW/c-ori (**c**). Data show the mean diarrhea scores per group.

A schematic of the serial passages in suckling mice and MA104 cells is indicated in Figure 1a. Mouse infections were performed as previously described [22,23,24,25,26]. Pregnant BALB/cCrSlc mice (gestation day 16) were purchased commercially (Japan SLC Inc., Shizuoka, Japan), housed individually, and delivered pups at 19.5 days of gestation on average.

#### 2.2.1. In Vivo Passaging of EW/v-ori

Six-day-old suckling mice (*n* = 6) were orally inoculated with 50 μL of EW/v-ori and monitored daily for 3 days for diarrhea by gentle abdominal palpation. At 3 days post-inoculation, pups were euthanized and colons were collected individually. Colons were homogenized in 400 μL PBS (+) (pH 7.5; 0.5 mM MgCl_2_, 1 mM CaCl_2_) using a BioMasher II (Nippi, Tokyo, Japan) and clarified by low-speed centrifugation to remove debris. Supernatants were transferred to microtubes, and equal volumes (15 μL) from each individual supernatant were pooled. To reduce unwanted gene rearrangements in vivo, believed to be favored under conditions of high viral input [27,28], the pooled supernatant—without filtration—was diluted 20-fold in serum-free Eagle’s minimum essential medium (MEM; Nissui, Tokyo, Japan) (incomplete medium), and 50 μL of the diluted inoculum was administered to each mouse in the next group (*n* = 6–8). EW/v-ori was passaged three times in suckling mice (EW/v-1 to EW/v-3).

#### 2.2.2. In Vitro Passaging of EW/c-ori in MA104 Cells

For the initial infection, EW/v-ori was activated with trypsin (type IX; porcine pancreas; 10 μg/mL; Sigma-Aldrich, Tokyo, Japan) and propagated in MA104 cells in incomplete medium containing trypsin (1 μg/mL). Infected cells were lysed by two freeze–thaw cycles. Lysates were then re-activated with trypsin (10 μg/mL) and inoculated onto fresh MA104 cells. EW/v-ori was passaged 10 times in MA104 roller-tube cultures to generate the cell-culture–adapted virus population EW/c-ori [25,29,30]. After passage 5, lysates were diluted 10^−1^–10^−2^ before the next propagation to reduce gene rearrangements.

#### 2.2.3. In Vivo Passaging of EW/c-ori

Because EW/c-ori was cell-culture adapted, titers could—in contrast to EW/v-ori—be determined by plaque assay. Six-day-old suckling mice were orally inoculated with 2 × 10^3^ p.f.u. of EW/c-ori and monitored daily for 3 days as described above. EW/c-ori was serially passaged six times in suckling mice (EW/c-1 to EW/c-6) using the same colon homogenization, pooling, and 20-fold dilution procedure described above.

#### 2.2.4. Diarrhea Scoring

The suckling mice were inspected daily for characteristics of diarrhea by gentle abdominal palpation for eight days. Rating of diarrhea (diarrhea score) was performed based on the following scale: 0, no stool; 1, soft orange stool; 2, soft mucous stool; and 3, liquid stool. In this study, the “diarrhea” status was defined as diarrhea score: ≥1 [23,24].

### 2.3. Viral Genomic RNA Extraction, cDNA Library Synthesis, and Next-Generation MiSeq Sequencing

Construction of a cDNA library and Illumina MiSeq sequencing for EW/c-ori and EW/v-ori were conducted as described previously [31,32]. Viral genomic dsRNAs from EW/c-ori or EW/v-ori were directly extracted using a QIAamp Viral RNA Mini Kit (Qiagen, Venlo, The Netherlands). In brief, a 200 bp fragment cDNA library ligated with bar-coded adapters was synthesized using an NEBNext Ultra RNA Library Prep Kit for Illumina v1.2 (New England Biolabs, Tokyo, Japan) according to the manufacturer’s instructions. The cDNA library was isolated using Agencourt AMPure XP magnetic beads (Beckman Coulter, Tokyo, Japan). After evaluating the quality and quantity of the purified cDNA library, nucleotide sequencing was performed on an Illumina MiSeq sequencer (Illumina, Tokyo, Japan) using a MiSeq Reagent Kit v2 (Illumina) to generate 151 paired end reads. MiSeq sequence data were analyzed using a CLC Genomics Workbench v8.0.1 (CLC Bio, Tokyo, Japan). Contigs were assembled from the obtained reads, after trimming, by de novo assembly. Using the assembled contigs as query sequences and the Basic Local Alignment Search Tool (BLAST, https://blast.ncbi.nlm.nih.gov/Blast.cgi, Blast+2.13.0, accessed on 14 March 2023) for searching the non-redundant nucleotide database of the National Center for Biotechnology Information (NCBI; https://www.ncbi.nlm.nih.gov/, accessed on 14 March 2023), it was determined which contigs represented the full-length nucleotide sequence for each segment of EW/c-ori or EW/v-ori including typical RVA segment termini. Nucleotide sequences were translated into amino acid sequences using GENETYX v17 (GENETYX, Tokyo, Japan). The proportion of individual nucleotides at amino acid mutation sites, summarized in Table 2, was investigated using Miseq data (mapping coverage vote).

### 2.4. Nucleotide Sequence Accession Numbers

Sequences determined by MiSeq for EW/c-ori—and for EW/v-ori if different from previously reported EW sequences—were deposited in the DDBJ and EMBL/GenBank databases. Accession numbers for EW/v-ori NSP1-NSP5, VP1-VP4, VP6, and VP7, genomic segments are LC909690-909700. Accession numbers for EW/c-ori VP1-VP4, VP6, VP7, and NSP1-NSP5 genomic segments are LC888974-888984.

### 2.5. RNA Extraction, RT-PCR, and Nucleotide Sequencing

To monitor the emergence of the candidate substitutions during in vivo passaging, we performed targeted RT-PCR and Sanger sequencing of selected segments from individual mouse colonic homogenates. The QIAamp Viral RNA Mini Kit was used to extract viral genomic dsRNAs individually from colonic homogenates after debris was removed by low-speed centrifugation. The dsRNAs were reverse transcribed using ReverTra Ace (Toyobo, Tokyo, Japan). The resulting complementary DNAs (cDNAs) were amplified using specific primers and KOD-Fx-NEO (Toyobo, Tokyo, Japan), and then the nucleotide sequences of the NSP4, VP2, VP4, and VP7 segments were verified by traditional Sanger sequencing (Eurofins Genomics, Tokyo, Japan).

### 2.6. Construction of Rescue EW T7 Plasmids Representing EW/v-ori and EW/c-ori

To enable segment-level testing of EW determinants, we constructed eight T7 plasmids representing those four EW gene segments that differed between EW/v-ori and EW/c-ori (VP2, VP4, VP7, and NSP4), one plasmid per gene segment. The eight T7 plasmids were synthesized by Eurofins Genomics or GeneScript (Tokyo, Japan), using a pUC57 vector (2710 bp) or a pEX-A2J2 vector (2659 bp) as vector backbones, resulting in the full-length segments being flanked by the T7 RNA polymerase promoter and HDV ribozyme sequences [33], followed by the T7 RNA polymerase terminator sequence [20]. The plasmids were designated as pT7/VP2EW, pT7/VP2EW_L71F, pT7/VP4EW, pT7/VP4EW_S470L_T612A, pT7/VP7EW, pT7/VP7EW_S12F_T75P, pT7/NSP4EW, and pT7/NSP4EW_T45M_H47R.

### 2.7. Reverse Genetics System

The protocol was basically as described previously [20,25,29,30]. The above-described rescue T7 plasmids were employed. A set of 11 rescue T7 plasmids for SA11-L2 virus had been prepared in our laboratory [21], namely, pT7/VP1SA11, pT7/VP2SA11, pT7/VP3SA11, pT7/VP4SA11-∆PstI, pT7/VP6SA11, pT7/VP7SA11, pT7/NSP1SA11, pT7/NSP2SA11, pT7/NSP3SA11, pT7/NSP4SA11, and pT7/NSP5SA11 [20]. In brief, the procedure was performed as follows. Monolayers of BHK/T7-9 cells in 6-well plates (Falcon, Bedford, MA, USA) were cotransfected with 11 T7 plasmids, representing the cloned cDNAs of 11 RVA dsRNA segments, in the following different quantities: (1) pT7/VP1SA11 (0.75 μg); (2) pT7/VP2SA11 (0.75 μg), pT7/VP2EW (0.75 μg), or pT7/VP2EW-L71F (0.75 μg); (3) pT7/VP3SA11 (0.75 μg); (4) pT7/VP4SA11-∆PstI (0.75 μg), pT7/VP4EW (0.75 μg), or pT7/VP4EW-S470L-T612A (0.75 μg); (5) pT7/VP6SA11 (0.75 μg); (6) pT7/VP7SA11 (0.75 μg), pT7/VP7EW (0.75 μg), or pT7/VP7EW-S12F-T75P (0.75 μg); (7) pT7/NSP1SA11 (0.75 μg); (8) pT7/NSP2SA11 (2.25 μg); (9) pT7/NSP3SA11 (0.75 μg); (10) pT7/NSP4SA11 (0.75 μg), pT7/NSP4EW (0.75 μg), or pT7/NSP4EW-T45M-H47R (0.75 μg); and (11) pT7/NSP5SA11 (2.25 μg). To generate recombinant SA11-L2-based single or double reassortant viruses containing one or two segments from EW/v-ori or EW/c-ori, the rescue T7 plasmid encoding the respective segment of SA11-L2 virus was exchanged with a rescue T7 plasmid corresponding dsRNA segment of EW(-derived) virus. This process was carried out for 4 of the 11 individual dsRNA segments. Following a 1-day incubation, the transfected BHK/T7-9 cells were washed with incomplete medium and then cocultured with overlaid CV-1 cells (1–5 × 10^5^ cells/mL) for 5 days in incomplete medium containing trypsin (0.9 μg/mL). After this, the cultures were subjected to two cycles of freezing and thawing and then treated with trypsin (10 μg/mL) for RV activation, followed by inoculation onto MA104 cells in a roller-tube culture [34]. After one additional passage in such a culture and several days incubation, recombinant viruses were rescued and—in case of the single reassortants—subsequently plaque purified in CV-1 cells, as previously described [35].

### 2.8. Electrophoretic Analysis of Viral Genomic dsRNAs

Viral genomic dsRNAs were extracted from cell cultures using a QIAamp Viral RNA Mini Kit (Qiagen). The extracted viral genomic dsRNAs were subjected to polyacrylamide gel electrophoresis (PAGE) analysis: they were run in a 10% polyacrylamide gel for 17 h at 19 mA at room temperature, followed by silver staining [36] to visualize the genomic dsRNA migration profiles.

### 2.9. Plaque Assay

The plaque assays were conducted as described previously [37]. Briefly, confluent monolayers of CV-1 cells in 6-well plates (Thermo Fisher Scientific, Rochester, NY, USA) were infected with trypsin-pretreated RVs, washed twice with incomplete medium, and then cultured with trypsin (1 μg/mL) in primary overlay medium (0.7% agarose). After 2 days, the cells were stained with secondary overlay medium containing 0.005% neutral red (Sigma-Aldrich) and 0.7% agarose. On the following day, plaque sizes were determined by measuring the mean diameters of 30 plaques in 2 independent assays. The virus titer from plaque-forming unit (pfu) assays was expressed as p.f.u./mL.

### 2.10. Focus-Forming Assays

Monolayers of MA104 cells in 96-well plates (Thermo Fisher Scientific, Rochester, NY, USA) were individually infected in triplicate with each recombinant virus that had been preactivated with 1 μg/mL of trypsin for 1 h at 37 °C. After washing the infected cell once with incomplete medium, the plate was incubated for 18–20 h. After washing the cell twice with PBS (+), the cells were fixed with cold methanol for 5 min. The methanol was completely removed and cells were incubated for 1 h at room temperature with rabbit antiserum specific for RV (1:50) (laboratory-produced) diluted in PBS (+). After washing the cells with PBS (+), cells were incubated for 1 h at room temperature with FITC-conjugated anti-rabbit IgG goat serum (1:50) (MP Bio Japan K.K., Tokyo, Japan) diluted in PBS (+). After washing the cells with PBS (+), the number of foci were counted under an Observer Z1 fluorescence microscope (Zeiss, Aichi, Japan), and the virus titer from focus-forming unit (f.f.u.) assays was expressed as f.f.u./mL.

### 2.11. Viral Growth

Monolayers of MA104 cells in 12-well plates (Thermo Fisher Scientific, Rochester, NY, USA) were infected in triplicate with trypsin-pretreated recombinant viruses at a multiplicity of infection (MOI) of 0.01, washed twice with incomplete medium, and then incubated in incomplete medium with trypsin (1 μg/mL) for 36 h. The infected cells were frozen and thawed twice before the measurement of viral titers by focus-forming assays.

### 2.12. Mouse Experiment for Recombinant Viruses

Five-day-old suckling mice from pregnant BALB/cCrSlc mice were orally administered 100 μL of cell culture supernatant containing each recombinant virus (8.0 × 10^4^ f.f.u./mouse) or incomplete medium as a control, and were then monitored daily over a period of 8 days for diarrhea following gentle abdominal palpation (as described above) and their body weight was measured.

### 2.13. Structural Visualization and Composite Rendering

Cryo-EM reconstructions of partial rhesus rotavirus particles (PDB IDs; 9C1H, 9C1J, 9C1L, 6WXE, 6WXF, 6WXG, 7UMS, 7UMT, and 8BP8) were investigated and some findings were visualized using PyMOL (version 3.0; Schrödinger, LLC, New York, NY, USA). For Figure 7, composite renderings were generated by superposing 9C1H and 9C1L or 9C1J and 9C1L. Residue numbering was based on the positions of the matching residues in the corresponding EW segments as sequenced in this study (EW-based residue numbering).

## 3. Results

### 3.1. Virulent and Attenuated EW After Bidirectional Passaging

The virulent murine EW stock (EW/v-ori) was serially passaged in suckling mice (EW/v-1 to EW/v-3; *n* = 6 per group) and consistently induced diarrhea beginning on day 1 post-inoculation, confirming its virulence (Figure 1a,b). In parallel, EW/v-ori was adapted to cell culture by 10 sequential passages in MA104 cells to generate the cell-culture–adapted population EW/c-ori, which was then serially passaged in suckling mice (EW/c-1 to EW/c-6; *n* = 4–8 per passage) (Figure 1a). During in vivo passaging of EW/c-ori, diarrhea onset shifted from day 2 in early passages (EW/c-1 to EW/c-3) to day 1 in later passages (EW/c-4 to EW/c-6), with a corresponding increase in mean diarrhea scores across passages (Figure 1c). Notably, plaque purification was not performed at any step.

### 3.2. Sequence Analysis of EW/v-ori and EW/c-ori; EW/c-ori Regains EW/v-ori Features After In Vivo Passaging

Whole-genome sequencing of EW/v-ori and the cell-culture-adapted population EW/c-ori identified only seven acquired amino-acid differences, mapping to NSP4 (T45M, H47R), VP2 (L71F), VP4 (S470L, T612A), and VP7 (S12F, T75P), plus a silent nucleotide mutation in VP6 (Table 1).

**Figure 2:**
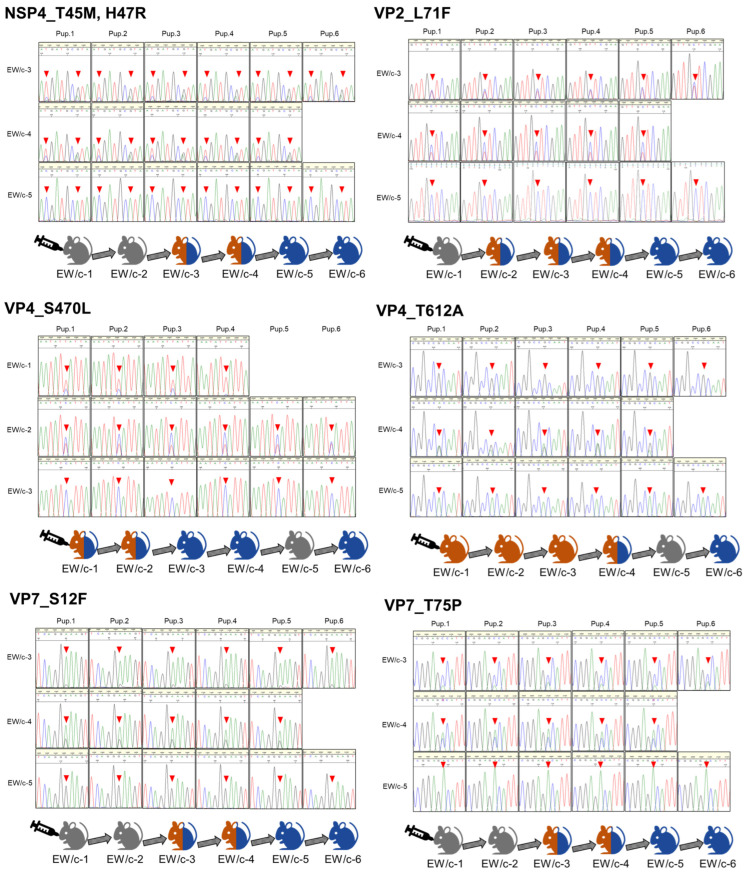
Reversion of EW/c-ori sequence changes during in vivo passaging. Targeted RT-PCR products spanning the seven amino-acid differences between EW/v-ori and EW/c-ori (NSP4, VP2, VP4, and VP7) were sequenced directly from individual colonic homogenates by Sanger sequencing. For each passage, the schematic summarizes when EW/v-ori-type versus EW/c-ori-type residues were detected based on inspection of chromatogram peaks. Orange and blue indicate samples dominated by EW/v-ori-type or EW/c-ori-type residues, respectively; orange/blue indicates mixed populations. Gray indicates no sequence data. The changes to the sequence during the serial passage are indicated by red arrowheads.

To track how these loci behaved during host readaptation, we sequenced targeted amplicons from individual mice across serial in vivo passages of EW/c-ori (EW/c-1 to EW/c-6, *n* = 4–8 for each passage). Across passages, the EW/v-ori–type residues increased and ultimately became dominant, consistent with reacquisition of an EW/v-ori(–like) genotype during in vivo selection (Figure 2). Notably, the VP4 S470 residue (EW/v-type) became dominant earlier than the other tracked loci (Figure 2). These experiments were conducted without plaque purification, and multiple nucleotides present at the substitution sites in EW/c-ori provided the variation source (Table 2).

### 3.3. Generation of Recombinant SA11-L2-Based Reassortants with One or Two Segments from EW

**Figure 3:**
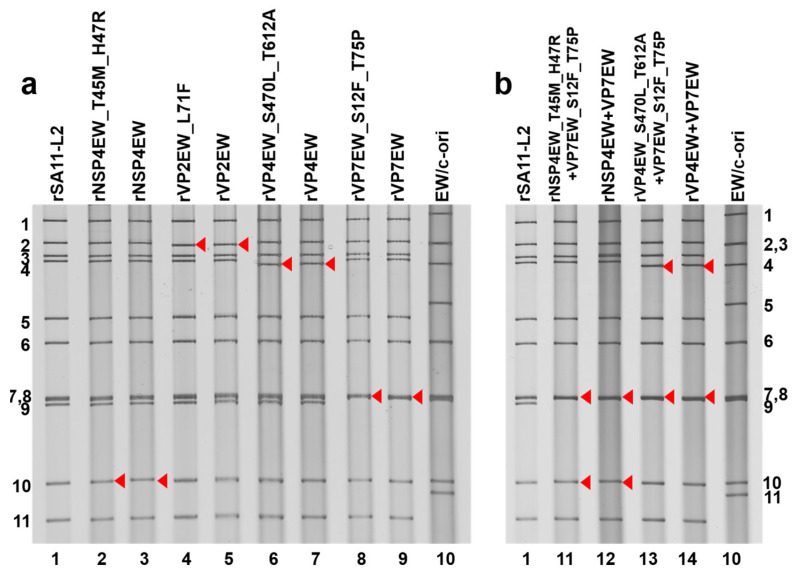
PAGE analysis of recombinant SA11-L2-based single or double reassortants containing EW (v- or c-type) gene segments. Lanes 1 and 10, dsRNAs from rSA11-L2 (lane 1) and EW/c-ori (lane 10). (**a**) Lanes 2–9, dsRNAs from rescued single reassortants: rNSP4EW_T45M_H47R (lane 2), rNSP4EW (lane 3), rVP2EW_L71F (lane 4), rVP2EW (lane 5), rVP4EW_S470L_T612A (lane 6), rVP4EW (lane 7), rVP7EW_S12F_T75P (lane 8) and rVP7EW (lane 9). (**b**) Lanes 11–14, dsRNAs from rescued double reassortants: rNSP4EW_T45M_H47R + VP7EW_S12F_T75P (lane 11), rNSP4EW + VP7EW (lane 12), rVP4EW_S470L_T612A + VP7EW_S12F_T75P (lane 13), rVP4EW + VP7EW (lane 14). Red arrowheads indicate the positions of the cDNA-derived EW segments. The numbers on the left and right indicate the orders of the genomic dsRNA segments of rSA11-L2 and EW/c-ori, respectively.

To determine the significance of each differentiated segment, we utilized an established 11 plasmid-only reverse genetics system to generate recombinant simian RV SA11-L2-based reassortants [20,29,30] with either one (short name “single reassortants”) or two (short name “double reassortants”) segments derived from EW/v-ori or EW/c-ori. The recombinant viruses were produced using a simian SA11-L2 genetic backbone consisting of 9 or 10 T7 plasmids that each encoded a different SA11-L2 dsRNA segment, plus either (i) one EW/v-ori segment (NSP4EW, VP2EW, VP4EW, or VP7EW), (ii) one EW/c-ori segment (NSP4EW_T45M_H47R, VP2EW_L71F, VP4EW_S470L_T612A, or VP7EW_S12F_T75P), (iii) two EW/v-ori segments (NSP4EW plus VP7EW, or VP4EW plus VP7EW), or (iv) two EW/c-ori segments (NSP4EW_T45M_H47R plus VP7EW_S12F_T75P, or VP4EW_S470L_T612A plus VP7EW_S12F_T75P). In PAGE analysis, the viral genomic dsRNAs extracted from the eight single reassortants (Figure 3a) and four double reassortants (Figure 3b) exhibited RNA migration patterns consistent with incorporation of the corresponding EW segment(s) into the SA11-L2 backbone. Nucleotide sequencing analysis of the extracted viral dsRNAs from single- and double-reassortants validated the authenticity of the individual manipulated genomic segments.

As a note regarding our strategy for generating double reassortants, preliminary data suggested that among the single reassortants, rVP7EW conferred the most pronounced virulence phenotype relative to rVP7EW_S12F_T75P in vivo. Therefore, to test whether the other analyzed segments could confer additive effects, we attempted to generate three double reassortants each for EW/v-ori and EW/c-ori, harboring their respective VP7 segment plus either their NSP4, VP2, or VP4 segment. However, despite multiple attempts, neither for EW/v-ori nor for EW/c-ori, the double reassortants introducing both VP7 and VP2 could be rescued. Thus, our final panel consisted of the eight single reassortants and four double reassortants described above.

### 3.4. In Vitro Characterization of the Eight Single Reassortants and Four Double Reassortants

**Figure 4:**
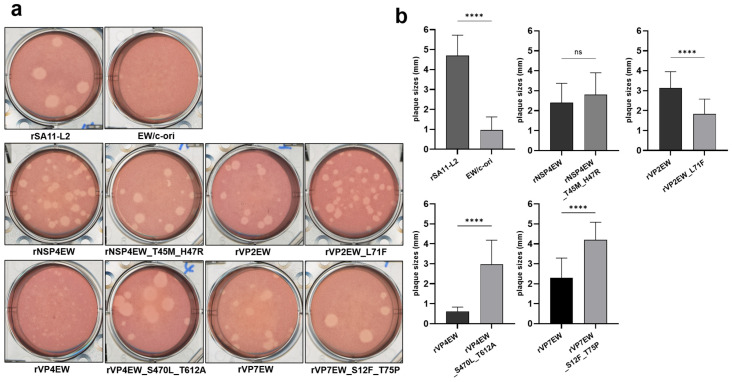
Plaque formation and plaque size by single reassortants in CV-1 cells. (**a**) Plaque formation by rNSP4EW, rNSP4EW_T45M_H47R, rVP2EW, rVP2EW_L71F, rVP4EW, rVP4EW_S470L_T612A, rVP7EW, rVP7EW_S12F_T75P, rSA11-L2, and EW/c-ori following direct plating on CV-1 cell monolayers. Experiments were repeated three times, and representative results are shown. (**b**) Plaque diameters were measured for 30 plaques per virus and are shown as means ± S.E. ****, *p* < 0.0001; ns, not significant.

Plaque sizes generated in CV-1 cell monolayers were compared after infection with the eight single reassortants, four double reassortants, rSA11-L2, EW/v-ori, and EW/c-ori at an MOI of 0.01 p.f.u/cell (Figure 4a). Compared to rSA11-L2, EW/c-ori formed much smaller plaques (Figure 4a,b), while for EW/v-ori no plaques were observed. In agreement with adaptation to cell culture of EW/c-ori, the single reassortants rVP4EW_S470L_T612A and rVP7EW_S12F_T75P formed much bigger plaques (diameter [mean ± S.E.]: 2.97 ± 1.22 mm and 4.20 ± 0.89 mm) than those for their respective EW/v-ori equivalents, rVP4EW and rVP7EW (0.62 ± 0.22 mm and 2.30 ± 0.99 mm) (Figure 4b). On the other hand, rNSP4EW_T45M_H47R formed similar size plaques as rNSP4EW (2.80 ± 1.10 mm and 2.40 ± 0.97 mm). Unexpectedly, rVP2EW_L71F induced smaller-sized plaques (1.83 ± 0.75 mm) than rVP2EW (3.13 ± 0.82 mm). Despite multiple attempts, plaques were not observed for any of the four double reassortants.

**Figure 5:**
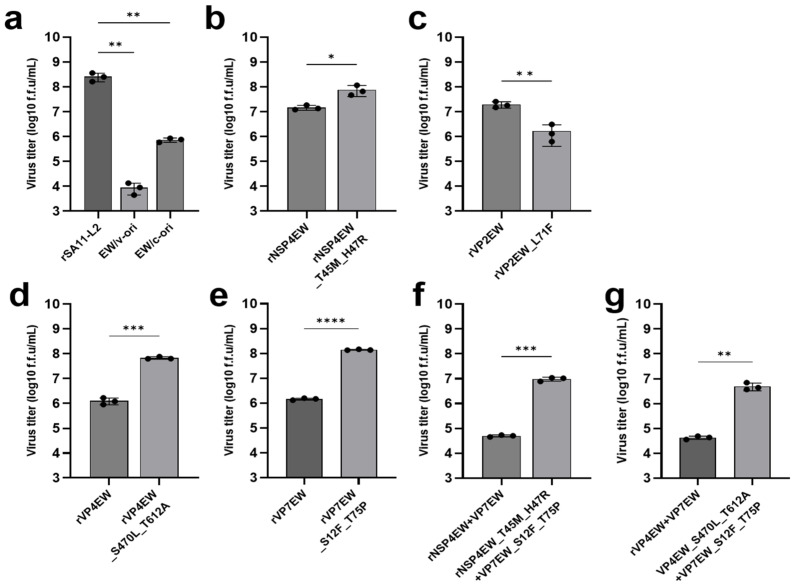
Growth properties of single and double reassortants. MA104 cells were infected with (**a**) rSA11-L2, EW/v-ori and EW/c-ori, (**b**–**e**) the single reassortments or (**f**,**g**) the double reassortments at an MOI of 0.01 and incubated for 36 h. The viral titers in the cultures were quantified by focus-forming assay after infection of fresh MA104 monolayers and are shown as the mean ± SD from three independent cultures (f.f.u./mL). ****, *p* < 0.0001; ***, *p* < 0.001; **, *p* < 0.01; and *, *p* < 0.1 (as calculated by unpaired *t*-test or ANOVA with Dunnett’s multiple comparisons test, as appropriate).

Viral titers of the different viruses were determined 36 h after infection of MA104 cells at an MOI of 0.01 f.f.u/cell, using a focus-forming assay for quantification (Figure 5). The titer of rSA11-L2 was 351-fold higher than that of EW/c-ori, which was 82-fold higher than that of EW/v-ori—the latter agreeing with cell-adaptation of EW/c-ori (Figure 5a). Comparing between the single reassortments with EW/v-ori versus EW/c-ori segment variants, rNSP4EW_T45M_H47R showed 5-fold higher titers than rNSP4EW (Figure 5b), rVP4EW_S470L_T612A showed 55-fold higher titers than rVP4EW (Figure 5d), and rVP7EW_S12F_T75P showed 96-fold higher titers than rVP7EW (Figure 5e); these observations suggest that mutations in NSP4, VP4, and VP7, all contributed to the cell-adaptation of EW/c-ori. In contrast, rVP2EW_L71F showed much lower titers than rVP2EW (Figure 5c), which is consistent with the plaque size measurements (Figure 4). The double reassortants rNSP4EW_T45M_H47R + VP7EW_S12F_T75P and rVP4EW_S470L_T612A + VP7EW_S12F_T75P showed 194-fold and 117-fold higher titers than their EW/v-ori-derived counterparts (Figure 5f,g). Comparison of the double reassortants with their matching single reassortants revealed an additive titer-reducing effect of introducing a second EW (v- or c-type) segment in the rSA11-L2 background, as each double reassortant yielded lower titers than either of its corresponding single reassortants (Figure 5); however, we did not investigate mixed v/c double reassortants, and the specific influence of the v → c substitutions on this effect cannot be properly assessed.

### 3.5. In Vivo Characterization of the Eight Single Reassortants and Four Double Reassortants

**Figure 6:**
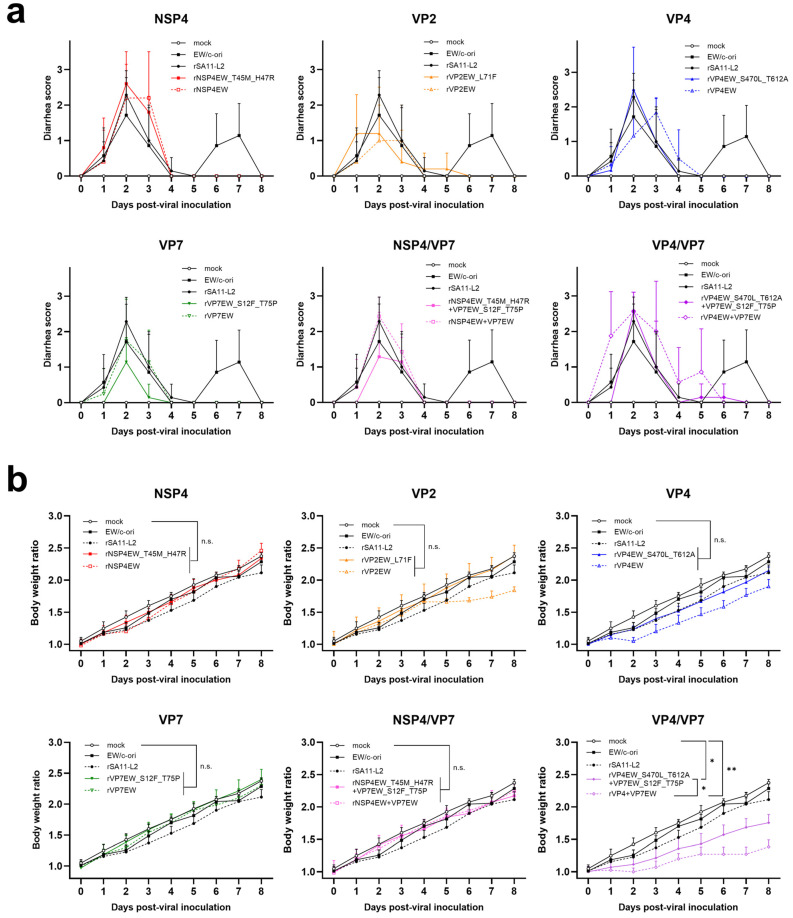
Pathogenicity of single and double reassortants, rSA11-L2 and EW/c-ori in suckling mice. Five-day-old suckling mice were orally administrated 100 μL of cell-culture supernatant containing each recombinant virus (8.0 × 10^4^ f.f.u./mouse) or incomplete medium as a mock control. (**a**) Diarrhea scores were assessed daily for 8 days using gentle abdominal palpation individually. (**b**) Body weights were measured daily in individual mice for 8 days and are shown as group averages normalized to the average body weight of the same group on day 0, which was set to 1.0. **, *p* < 0.01; and *, *p* < 0.1 (as calculated by unpaired *t*-test or ANOVA with Dunnett’s multiple comparisons test, as appropriate). “n.s.” indicates not significant. In all the figures, the data for mock, EW/c-ori and rSA11-L2 is the same (**a**,**b**).

To evaluate the impact of the recombinant virus in vivo, 5-day-old suckling mice were orally inoculated with eight single reassortants, four double reassortants, EW/c-ori, or rSA11-L2 at a dose of 8.0 × 10^4^ f.f.u./mouse, or mock inoculated. The mice were monitored daily over eight days for diarrhea severity (Figure 6a) and body weight (Figure 6b).

As for diarrhea, among the matched EW/c-type versus EW/v-type single-reassortant pairs, only the rVP7 pair showed consistent differences during all investigated days with rVP7EW_S12F_T75P inducing less severe diarrhea than rVP7EW. Within matched EW/c-type versus EW/v-type double reassortant pairs, the suckling mice administered rVP4EW_S470L_T612A + VP7EW_S12F_T75P or rNSP4EW_T45M_H47R + VP7EW_S12F_T75P exhibited less severe diarrhea than the matching EW/v-ori-based double reassortants. We can only speculate as to why EW/c-ori showed two diarrhea-score peaks in suckling mice, but similar findings have been reported before for suckling mice infected with murine RV [18,38].

In regard to body weight, the groups infected with the single reassortants rVP2EW_L71F or rVP4EW_S470L_T612A, or the double reassortant rVP4EW_S470L_T612A + VP7EW_S12F_T75P showed a higher weight gain than the groups infected with the matching EW/v-ori-based reassortants; however, for this observation, only the rVP4/VP7 double reassortants showed statistical significance.

## 4. Discussion

In this study, the four segments NSP4, VP2, VP4, and VP7 of virulent murine RVA strain EW (“EW/v-ori”) together showed 7 amino acid mutations (Table 1) after 10 passages in cell culture (generating “EW/c-ori”). After reintroducing EW/c-ori into mice, each of these mutated segments was negatively selected against their wild-type counterparts (Figure 1 and Figure 2). The process showed a strong selection with a large degree of homogeneity among the multiple mouse individuals per passage (Figure 2), with the observed pattern being possible because EW/c-ori was a mixed (not plaque-purified) population with the original EW/v-ori-type variants and intermediate variants still present (Table 2). In vitro phenotype comparisons of the cell-adapted versus wild-type segments—after their introduction into a reverse genetics simian RVA infectious system—showed that each of the mutated segments NSP4, VP4, and VP7 provided significant growth advantages in cell culture (Figure 4 and Figure 5). However, for VP2 the opposite was observed (Figure 4 and Figure 5), which we speculate might be due to the mutation having a context-dependent negative effect within the simian RVA recombinant system. Upon in vivo analysis of the segments, only the rVP4/VP7 double reassortants showed reductions in virulence with statistical significance in association with the cell-culture-adapted (EW/c-ori) mutations (Figure 6), which agrees with other studies suggesting the need of cumulative cell-culture-adapted differences for finding a significant reduction in disease severity (e.g., [13,18,39,40,41,42,43]). Regarding this issue, it should be realized that the “attenuation” system selects for improved replication in cell culture, which not necessarily needs to have a dramatic impact in vivo.

After reintroduction of the cell-culture-adapted population into mice, VP4 residue S470 became dominant more rapidly—already by passage 3—than any of the other six variable sites (Figure 2). This pattern supports an independent in vivo selection of S470 at the single-residue level, whereas for the remaining sites we cannot exclude co-selection or passive hitchhiking of one mutation with another (particularly within NSP4 and VP7). Nevertheless, the highly consistent allele-frequency shifts across mice (Figure 2) argue against stochastic drift as the sole explanation and indicate selection on all four mutated segments at least at the segment level; in addition to VP4 S470, residue-level selection can also be inferred for VP4 T612, which rose homogeneously in frequency later than VP4 S470 during in vivo passaging (Figure 2).

Although cell-culture-adapted live-attenuated RVA strains are successfully used as vaccines, the molecular mechanism of RVA attenuation is not well understood. As summarized in Supplementary Table S1, there have been quite a number of studies that compared the sequences between virulent wild-type and attenuated RVA strains. As shown in Table 3 (a selection of the data shown in Supplementary Table S1), among the seven amino acid mutations we found, in four cases similar mutations were reported before, sometimes involving different RVAs and adaptation to different cell lines: NSP4 T45(A/I/M), VP4 S470(F/H/L), VP4 T612A, and VP7 (V/I/T)75(T/I/M/L). Association does not necessarily imply a causal relation, and studies aiming to find such causal relation—for example by using reverse genetics infectious systems—are far fewer [18,44,45,46]. To the best of our knowledge, none of the seven mutations we found has previously been implicated individually in attenuation/virulence, but Kawagishi et al. [18] demonstrated an in vivo impact of the 2-amino acid motifs VP4_D452N_S470L and VP4_T612A_A711T (they were not able to discern whether one or two of the amino acid changes contributed). The present study appears to be the first to deduce the relevance of the S470L mutation at the single residue level.

**Figure 7:**
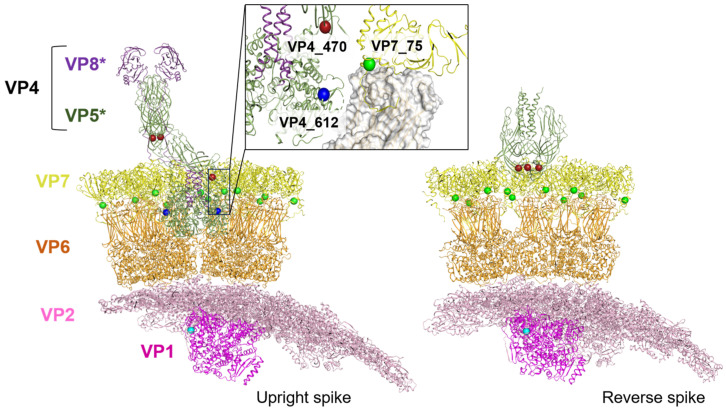
Structural context of EW capsid substitutions in upright and reversed VP4 spike conformations. Partial cutaway views of a triple-layered simian RVA particle are shown with the VP4 spike in the upright conformation ((**left**); composite of PDB 9C1H and 9C1L) or the reversed/folded-back conformation ((**right**); composite of PDB 9C1J and 9C1L). VP4 is displayed as its proteolytically cleaved domains VP8* (purple) and VP5* (green); VP7 is shown in yellow, VP6 in orange, VP2 in light pink, and VP1 in magenta. EW residues mapped as spheres are VP2-71 (cyan), VP4-470 (red), VP4-612 (blue), and VP7-75 (green). The boxed region is enlarged (inset) to highlight the spatial relationship of VP4-470, VP4-612, and VP7-75 near intersubunit interfaces (VP6 shown as surface in the inset for clarity). The VP7 N-terminal region containing residue 12 was not resolved in these structures.

While avoiding overdiscussion, we would like to shortly discuss the seven mutations found in this study at the protein structural level, with an emphasis on VP4 S470L. NSP4 has been described as a viroporin implicated in Ca^2+^ dysregulation and residues 45 and 47 are positioned at the cytosolic boundary of the H2 transmembrane region, adjacent to the proposed membrane-active/viroporin domain [47]; therefore, their mutations might affect NSP4’s viroporin activity. The VP2, VP4 (which is proteolytic cleaved into VP5 and VP8), and VP7 proteins are capsid proteins, and the locations of the residues VP2 71, VP4 470 and 612, and VP7 75 (the VP7 stretch with residue 12 was not structurally resolved in the structures we analyzed) are shown in Figure 7, which is based on published cryo-electron microscopy structures of a simian RVA.

**Figure 8:**
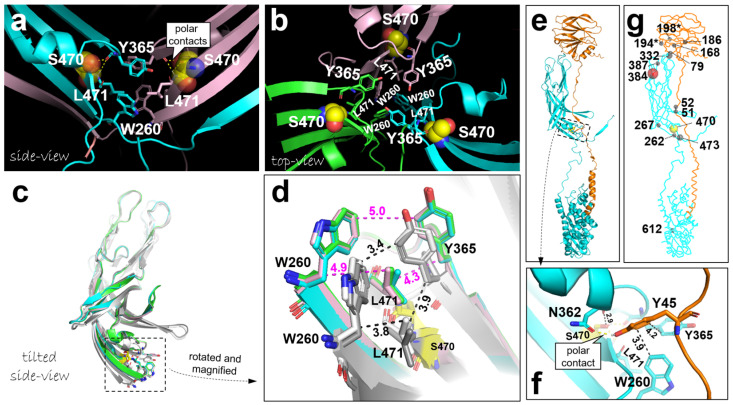
VP4 residue S470 appears to stabilize the upright spike conformation near the end of the β-barrel region, at the interface of multiple VP4 domains. (**a**) In the two VP4 molecules (cyan and pink) in an upright spike, S470 (shown in yellow as both sticks and transparent spheres) has a tendency to form polar contacts (dashed yellow lines) with the main chains of Y365 and L471, which together with W260 form a hydrophobic complex that interacts with the similar patch on the second VP4 molecule—such polar contacts were identified in four of the eight investigated upright VP4 molecules in PDB accessions 9C1H (here depicted), 6WXE, 7UMS, and 8BP8. (**b**) In contrast, in the three VP4 molecules in the reverse spike structure, S470 was not found to have this tendency, as none of the VP4 molecules in the reported reversed and intermediate spike configurations in PDB accessions 6WXF, 6WXG (here depicted), 7UMT, and 9C1J showed these polar contacts. (**c**,**d**) Superimposition of the segments W260-to-L471 of the two and three VP4 molecules shown in (**a**) (here grayish) and (**b**) (original colors), respectively, highlights compactness of the hydrophobic knob formed by L471, Y365, and W260. Dashed lines with numbers indicate distances in Å. (**e**,**f**) In a single VP4 molecule in the upright spike (VP5 in cyan, VP8 in orange), this structure connects with, and possibly stabilizes, the well-conserved VP4 residues Y45 and N362 that can make a VP5-VP8 interchain polar contact (PDB 9C1H). Notably, another mutation in this region, E262G, which causes enhanced replication in MA104 cells [45], may also affect interdomain interactions. (**g**) This figure is similar to (**e**) although in ribbon format, with Cα atoms shown as spheres of those positions that—according to Supplementary Table S1—were found associated with attenuation/virulence in two or more publications (representing independent studies; however, studies were not similarly sensitive for selecting and finding variants, and this approach should be considered a very rough estimation only); bigger spheres are used according to the number of studies, with special attention for position 384 (red; reported in 5 publications) and 470 (yellow; now reported in 4 publications). * Around positions 194 and 198 the alignment between sequences was inconclusive, making it difficult to pinpoint the exact positions in the EW sequence.

The figure shows that all the mutations are in proximity of intermolecular contacts, and they may affect such interactions. RVA virions are triple-layered particles with an inner VP2 core (that includes the genome and polymerase complex), a middle VP6 layer, and an outer capsid of VP7 studded with VP4 spikes; during entry, the VP4/VP7 outer layer is shed, delivering a transcriptionally active double-layered particle (DLP) into the cytosol [48]. In the intestinal lumen (or in vitro with trypsin), VP4 is proteolytically cleaved into VP8 (the primary attachment/receptor-binding domain of VP4) and VP5 (the membrane-interaction/penetration domain of VP4), which primes infectivity and prepares the spike for the entry-associated conformational transitions. These transitions include a VP5 “fold-back” (“reversed conformation”) that promotes membrane penetration, coupled to VP7 uncoating/decapsidation and release of the DLP. Possibly the mutations in VP4 and VP7 acquired in EW/c-ori accelerate the transitions between the stages, like the fold-back of VP5 and the shedding of the outer capsid; however, this is speculation only. In Figure 8, we discuss how the VP4 S470L mutation may reduce the stability of the spike if in the upright conformation and so, potentially, enhance membrane penetration. The trade-off between spike stability and readiness to undergo entry-associated rearrangements may differ between cell-culture and in vivo conditions.

Figure 8g is a rough approach to identify the VP4 regions with most variation in residues associated with cell culture- and/or in vivo-adaptation, based on our literature summary in Supplementary Table S1. The Cα atoms of residues at positions that were found substituted in two or more of the listed studies (publications) are shown as spheres, with the size of the sphere indicative of the number of studies. The figure shows that S470 is located in one of the regions that is frequently under selection, and the S470(F/H/L) mutation has now been identified in four different studies (Table 3 and Supplementary Table S1). The only VP4 position of which a substitution has more often been reported is position 384 (Supplementary Table S1 lists five studies), and by using a reverse genetics infectious system a D384H mutation was shown to reduce virus shedding in vivo [46].

Some of our experimental and/or literature findings for attenuation-associated residues highlighted above agree with RVA vaccine Rotarix^®^ (RIX4414; 89-12) (GlaxoSmithKline, Tokyo, Japan) sequences, as this live-attenuated vaccine acquired residues (numbering adjusted based on EW sequences) NSP4 I45 (GenBank WMB80880; [49] [Table 3 and Supplementary Table S1]), VP4 Y384 (WMB80874; [40] [Supplementary Table S1]), and VP7 F12 and I75 (WMB80876; precursor sequences unknown). This supports the practical relevance of our analyses.

**Table 3:** Summary of publications finding similar amino acid substitutions in relation to attenuation/virulence as the present study. An extensive literature review describing mutations in various RVA strains in association with attenuation/virulence revealed that four of the seven mutations were identical or similar to previous findings. For a more complete analysis, see Supplementary Table S1. Residue positions and amino acids in blue relate to the corresponding EW sequences in our study. Vir., virulent/wild-type/in vivo-adapted; Atten., attenuated/cell-culture-adapted; Ref., Reference.

Topic	Strain	Gene Segment	Amino Acid Position	Vir.	Atten.	Ref.
Sequence comparison between NSP4 of virulent and cell culture-attenuated (vaccine) human RV	Human RV (89-12)	NSP4	45 (45)	T (T)	A (M)	[39]
Sequence comparison between NSP4 of wild-type and cell culture-attenuated murine RV	MurineRV (EW)	NSP4	45 (45)	T (T)	M (M)	[50]
	MurineRV (EHP)					
Sequence comparison between three wild type human G1P[8] RV strains derived from diarrheal stool samples before and after passaging in cell culture	Human G1P[8] RV (Wa, DC3695, DC5685)	NSP4	45 (45)	T (T)	A (M)	[51]
		VP4	471 (470)	S (S)	F (L)	
		VP7	75 (75)	V (T)	M (P)	
				I (T)	T/I/M (P)	
Sequence comparison between two virulent human (Wa, G1P[8] and M, G3P[8]) and two virulent porcine (Gottfried, G4P[6] and OSU. G5P[7]) RV strains and their cell-culture-adapted derivates	Human RV (Wa)	VP4	471 (470)	S (S)	H (L)	[41]
	Porcine RV (Gottfried)			S (S)	L (L)	
	Human RV (M)	VP7	75 (75)	T (T)	L (P)	
Sequences were determined of Rotarix vaccine-derived isolates from 30 rotavirus-positive infants with gastroenteritis to screen for potential regaining-of-virulence markers	Rotarix vaccine-derived isolates from rotavirus-positive infants	NSP4	45 (45)	T (T)	I (M)	[49]
VP4 sequence comparison between wild-type (EW) and cell-culture-adapted (ETD_822) murine RV, and using reverse genetics to assess them and their chimera for virulence. Especially an EW VP4 fragment including the differences D452 and S470 enhanced virulence	MurineRV (EW)	VP4	470 (470)	S (S)	L (L)	[18]
			612 (612)	T (T)	A (A)	

Although none of the individual methods in our study is new, we introduce a compact yet comprehensive system for finding mutations that drive attenuation (cell culture adaptation) versus in vivo virulence. Critical components of our system are:

- The use of a virulent wild-type murine RVA strain (EW in this case) and its natural host;
- Attenuation to cell culture with a limited number of passages (10 in this case) to limit the number of selected mutations;
- Reintroduction of the attenuated virus population—which is a mixed population because plaque purification is not performed—into the natural host;
- Full genome sequence analysis of the virulent and attenuated virus populations to identify differences, and targeted sequencing of the mutated regions during passaging in vivo. This investigates whether the adaptations to cell culture are true virulence markers and undergo a negative selection in vivo;
- Introduction of the segments of the virulent versus attenuated viruses into a plasmid-based reverse genetics infectious system to allow comparison of their phenotypes;
- Phenotyping in vitro (plaque formation, titer) and in vivo (diarrhea, body weight).

In future studies, we hope to also compare single-residue-mutated segments and use an EW-based reverse genetics infectious system to avoid interference of other factors. We also hope to compare more combinations of the different mutations to study additive effects. Additionally, we plan to investigate several of the highlighted attenuation/virulence markers by introducing them into the reverse genetics infectious system that we recently generated for the Rotarix^®^ vaccine strain RIX4414 [25], in order to test whether they can modify (enhance virulence or further attenuate)—and help explain—the strain’s attenuated phenotype.

## 5. Conclusions

This study introduces a system for efficiently identifying mutations associated with attenuation, underlines the relevance of VP4 and VP7 for virulence attenuation, firmly establishes—by comparison with previous studies—association with mutations NSP4 T45(A/I/M), VP4 S470(F/H/L), VP4 T612A, and VP7 (V/I/T)75(T/I/M/L/P), and suggests a role of VP4 S470 as a key determinant of virulence at the single residue level. These conclusions may inspire future strategies of RVA live vaccine development.

## Figures and Tables

**Table 1:** Summary of the nucleotide and amino acid substitutions identified between EW/v-ori and EW/c-ori. Whole-genome sequencing of EW/v-ori and EW/c-ori were sequenced by using an Illumina MiSeq sequencer. F: phenylalanine. L: leucine. S: serine. A: alanine. T: threonine. P: proline. R: arginine. H: histidine.

Gene Segment	Nucleotide Position	Amino Acid Position	Amino Acid	
			EW/v-ori	EW/c-ori
NSP4	174	45	T	M
	180	47	H	R
VP2	227	71	L	F
VP4	1418	470	S	L
	1843	612	T	A
VP7	83	12	S	F
	271	75	T	P
VP6	284	87	Y	Y

**Table 2:** The mapping coverage vote of EW/c-ori genome (A, C, G and T) from an Illumina MiSeq sequencer. The Illumina MiSeq sequence data on the identified seven amino acid sites indicates that the EW/c-ori contained a mixed population of nucleotides prior to administration to the mice.

Gene Segment	Nucleotide Position	Amino Acid Position	Ratio of Nucleotide of EW/c-ori (%)			
			A	C	G	T
NSP4	174	45	1.9	20.6	2.2	75.0
	180	47	28.3	1.7	67.4	2.4
VP2	227	71	0.6	44.3	1.0	54.1
VP4	1418	470	0.0	9.5	0.2	90.3
	1843	612	0.0	0.7	98.7	0.7
VP7	83	12	0.2	0.4	0.7	98.7
	271	75	0.5	99.1	0.2	0.1

## Data Availability

The nucleotide sequence data obtained in this study have been deposited in the DDBJ and EMBL/GenBank data libraries. Sequences determined by MiSeq for EW/c-ori—and for EW/v-ori if different from previously reported EW sequences—were deposited in the DDBJ and EMBL/GenBank databases. Accession numbers for EW/v-ori NSP1-NSP5, VP1-VP4, VP6, and VP7, genomic segments are LC909690-909700, respectively. Accession numbers for EW/c-ori VP1-VP4, VP6, VP7, and NSP1-NSP5 genomic segments are LC888974-888984, respectively.

## Supplementary Materials

The following supporting information can be downloaded at: https://www.mdpi.com/article/10.3390/v18070747/s1, Table S1: Publications that compared the NSP4, VP2, VP4, and/or VP7 sequences of related rotavirus A viruses based on differential acquisition of attenuation/virulence are summarized from our point of interest [18,28,39,40,41,42,44,45,46,49,50,51,52,53,54,55,56,57].
