# Supplementary material for: Identification of Murine Rotavirus Virulence Determinants Using Bidirectional Selective Passaging and a Reverse Genetics System
Source: Viruses. 2026 Jul 6;18(7):747. doi: 10.3390/v18070747 (PMC13431595; doi:10.3390/v18070747)
Supplement: Supplementary file 1 [file viruses-18-00747-s001.zip › viruses-4390717-supplementary.pdf]

Supplementary Table S1

Publications that compared the NSP4, VP2, VP4, and/or VP7 sequences of related rotavirus A viruses based on differential acquisition of attenuation/virulence are summarized from our point of interest

Residue positions and amino acids in blue relate to the corresponding EW sequences in our study. Vir., virulent/wildtype/*in-vivo* -adapted; Atten., attenuated/cell-culture-adapted; Regainded vir., regained virulence/*in vivo* adaptation.
Background colors are used to highlight similar findings to those in the present study; dots are added if the residue position is the same but the type of change different.

Topic	Method	Gene segment	Amino acid position	Vir.	Atten.	Regained vir.	Reference
Sequence comparison between NSP4 of virulent and cell culture-attenuated (vaccine) human RV (strain 89-12)	Attenuation: 26 passages in primary African green monkey kidney cells and 7 additional passages in an AGMK cell line. Sequencing: only NSP4. Functional analysis for segment or mutation: not performed.	NSP4	45 (45)	T (T)	A (M)		Ward *et al.* (1997) [39]
Sequence comparison between NSP4 of wild-type and cell culture-attenuated murine rotavirus strain EW.	Attenuation: unknown number of passages in AGMKcells and subsequently in MA104 cells. Sequencing: only NSP4. Functional analysis for segment or mutation: not performed.	NSP4	45 (45)	T (T)	M (M)		Angel *et al.* (1998) [50]
			173 (173)	A	T		
Sequence comparison between NSP4 of wild-type and cell culture-attenuated murine rotavirus strain EHP.		NSP4	45 (45)	T (T)	M (M)		
			140 (140)	R	S		
			174 (174)	P	S		
Sequence comparison between NSP4 of wild-type and cell culture-attenuated murine rotavirus strain EC.		NSP4	13 (13)	V	A		
			38 (38)	S	F		
			150 (150)	R	Q		
Sequenced comparison of NSP4 of two (Wa and M strains) virulent human RVs and their cell-culture-adapted derivates.	The Wa rotavirus was adapted to cell culture in monkey kidney cells (MA104) after 11 gnotobiotic pig passages, plaque purified and serially passaged 27 times in these cells. The virulent M strain was serially passaged in gnotobioticpiglets 7 times and the M strain was attenuated after plaque isolation and serial passage 46 times in MA104 cells. The NSP4 sequences were determined and compared between the pairs.	NSP4	(Wa) 13 (13)	V	A		Chang *et al.* (1999) [52]
			(Wa) 16 (16)	L	S		
			(Wa) 43 (43)	P	L		
			(M) 53 (53)	T	I		
			(M) 104 (104)	K	E		
Sequence comparison of NSP4 of RVA strains from both diarrheal and asymptomatically-infected kittens.	NSP4 sequences were compared between feline RVA isolates: 4 diarrhea-source isolates (FRV-1, FRV72, FRV73, FRV317), and 6 asymptomatic-source isolates (FRV64, FRV70, FRV303, FRV348, FRV381, FRV384).	NSP4	No consistent differences were found				Oka *et al.* (2001) [53]
Sequence comparison between VP4 of virulent and cell culture-attenuated (vaccine) human RV (strain 89-12).	See above (Ward RL *et al*. 1997). *We can't be sure how these VP4 positions relate to the numbering in our study as the article does not explain the numbering source.	VP4	51*	G	D		Ward *et al.* (2006) [40]
			167*	L	F		
			331*	S	F		
			385*	D	Y		
			695*	N	I		
Sequence comparison between cell culture-attenuated murine rotavirus strain EB and its derivates after alternating and variable passaging in mice or cell culture.	Bidirectional adaptation: alternating multiple serial passages in mice and AGMK and MA 104 cells. Sequencing: full genome of 9 viruses isolated from mice and 11 viruses isolated from cell culture. Here at the right we only show the variation found in at least three isolates, using small font if in only one or two of the respective isolates. Functional analysis for segment or mutation: not performed.	NSP4	21 (21)	L/i	L/I		Tsugawa *et al.* (2014) [28]
			37 (37)	V/A	V		
		VP2	123 (123)	I	I/L		
		VP4	311 (310)	V	V/I		
			385 (384)	E/K	E		
			470 (469)	I	I/T		
			537 (536)	L/f	F		
			538 (537)	D/G	D		
			558 (557)	T/a	A		
		VP7	104 (104)	Q	Q/H		
			119 (119)	K	K/E		
			238 (238)	N/D	N		
			309 (309)	A	A/T		
		(Wa) (HT29) NSP4	34 (34)	P	T (p20)		
			83 (83)	A	V (p40)		
		(Wa) (AGMK) NSP4	45 (45)	T (T)	A (p20) (M)		
			57 (57)	A	V (p40)		
		(*) (HT29) NSP4	38 (38)	S	A (p40)		
			50 (50)	S	L (p40)		
		(*) (AGMK) NSP4	45 (45)	T (T)	A (p20) (M)		
			108 (108)	R	R/C (p60)		
		(**) (HT29) NSP4	146 (146)	T	M (p20)		
		(**) (AGMK) NSP4	45 (45)	T (T)	A (p20) (M)		
			50 (50)	S	L (p20)		
			107 (107)	R	T (p40)		
			139 (139)	V	A (p40)		
			146 (146)	T	M (p20)		
		(Wa) (HT29) VP2	no change				
		(Wa) (AGMK) VP2	7 (7)	G	E (p40)		
		(*) (HT29) VP2	439 (423)	V	I (p20)		
			763 (747)	N	T (p40)		
		(*) (AGMK) VP2	218 (204)	S	S/T (p60)		
			387 (371)	Q	Q/R (p40)		
		(**) (HT29) VP2	878 (856)	V	I (p40)		
		(**) (AGMK) VP2	no change				
			79 (79)	D	G (p40)		
			187 (188)	S	G (p40)		

Sequence comparison between three wild type human G1P[8] RV strains (Wa, DC3695*, DC5685**) derived from diarrheal stool samples before and after passaging 20-40-60 times times in two different cell cultures. Most substitutions were detected in VP4, followed by VP7 and NSP4.	RV derived from diarrheal stool was passaged 20, 40, or 60x in HT29 or AGMK cells ("p" numbers here at the right indicate when first detected). Sequencing: full genome. Functional analysis for segment or mutation: not performed.	(Wa) (HT29) VP4	316 (315)	T	A (p20)		Tsugawa *et al.* (2016) [51]
			368 (367)	R	K (p20)		
			593 (592)	V	L (p60)		
			727 (726)	K	T (p60)		
		(Wa) (AGMK) VP4	193 (194)	deletion	K (40)		
			385 (384)	D	N (p20)		
			474 (473)	P	S (p20)		
		(*) (HT29) VP4	146 (147)	G	R (p20)		
			171 (172)	G	R (p20)		
			187 (186)	S	G (p40)		
			262 (261)	K	R (p40)		
		(*) (AGMK) VP4	52 (52)	H	N (p40)		
			385 (384)	D	N (p60)		
			471 (470)	S (S)	F (p40) (L)		
		(**) (HT29) VP4	206 (205)	I	V (p60)		
			220 (219)	N	T (p20)		
			267 (266)	N	D (p20)		
			339 (338)	S	L (p20)		
		(**) (AGMK) VP4	199 (198)	T	I (p60)		
			268 (267)	R	T (p20)		
			324 (323)	N	T (p20)		
			331 (330)	S	F (P20)		
			385 (384)	D	H (p40)		
			540 (539)	T	S (p60)		
		(Wa) (HT29) VP7	71 (71)	T	N (p20)		
			75 (75)	V (T)	M (p40) (P)		
			129 (129)	V	I (p20)		
			201 (201)	Q	Q/R (p60)		
		(Wa) (AGMK) VP7	no change				
		(*) (HT29) VP7	201 (201)	Q	R (p20)		
			202 (202)	T	M (p20)		
		(*) (AGMK) VP7	75 (75)	I (T)	T/I/M (p60) (P)		
			302 (302)	Y	H (p40)		
			316 (316)	A	A/V (p60)		
		(**) (HT29) VP7	71 (71)	T	I (p40)		
			74 (74)	G	E (p60)		
			201 (201)	Q	R (p40)		
		(**) (AGMK) VP7	74 (74)	G	E (p40)		
Testing the effect in vitro and in vivo of a single residue VP4 mutation R446G, located in a receptor binding motif, using a rhesus rotavirus (RRV) reverse genetics system. The mutation attenuated both in vitro and in vivo phenotypes.	RRV (RRVVP4-R446G) with or without a single amino acid change (R446G sitiuated in the VP4 three-aa motif SRL that the authors had previously determined as important for receptor binding), produced through reverse genetics, were compared for cholangiocyte tropism in vitro and pathogenicity in neonatal mice.	VP4	Rescued RVV strains with G446 (444) instead of wildtype R446 showed reduced cholangiocyte binding and infectivity, and reduced virulence in neonatal mice.				Mohanty *et al.* (2017) [44]
Sequence comparison of the VP7 gene in eight human recipients who received the Rotarix vaccine one month prior and kept shedding virus longer than average, thereby identifying potentially virulence enhancing mutations.	Eight infants (numbered 2, 9, 10, 17, 19, 35) received Rotarix vaccince and shed virus in their stool for at least one month. Changes in VP7 sequence were monitored. Only in patients 2 and 19 aa changes persisting until 1 month were observed.	VP7	(No. 2) 151 (151)		D	N	Kaneko *et al.* (2017) [54]
			(No. 2) 280 (280)		Q	R	
			(No. 19) 123 (123)		S	N	
Virulent human rotavirus strain CDC-9 was adapted to cell culture, resulting in 6 aa substitutions in VP4, and 1 each in VP1, VP6, NSP1, and NSP5.	CDC-9 RV was passaged 12x in MA104 cells and then until passage 44 in Vero cells. Sequencing: full genome. Functional analysis for segment or mutation: not performed. Although this article did not specify the changes except for the positions, they were explained in the Bessey *et al*. 2025 article by the same group. Red boxing in he columns at the right relates to functional relevance determined in that 2025 study.	VP4	51 (51)	G	D (p12)		Resch *et al.* (2020) [42]
			331 (330)	S	F (p12)		
			364 (363)	M	I (p25)		
			385 (384)	D	H (p25)		
			388 (387)	I	L (p28)		
			498 (497)	D	N (p48)		
		(Wa) NSP4	16* (16)	L	S		
			34* (34)	P	L		
			40* (40)	V	A		
		(M) NSP4	no change				
		(Go) NSP4	no change				
		(OSU) NSP4	no change				
		(Wa) VP2	no change				
		(M) VP2	no change				
		(Go) VP2	650 (638)	S	L		
			875 (863)	P	S		
		(OSU) VP2	no change				
		(Wa) VP4	51 (51)	G	V		
			131 (131)	R	S		
			385 (384)	D	N		
			471 (470)	S (L)	H (L)		
			668 (667)	P	L		

Sequence comparison between two virulent human (Wa, G1P[8] and M, G3P[8]) and two virulent porcine (Gottfried, G4P[6] and OSU. G5P[7]) RV strains an their cell-culture-adapted derivates.	Wa, M, Gottfried ("Go"), and OSU strains maintained in gnotobiotic piglets for 22, 11, 12 and 9 serial passages, respectively, with their attenuated counterparts serially passaged in MA-104 cell cultures for 25, 43, 54 and 43 passages, respectively. Sequencing: full genome. Functional analysis for segment or mutation: not performed. *The authors seem to have made mistakes in numbering the Wa NSP4 aa positions, which we adjusted here.	(M) VP4	77 (77)	P	L		Guo *et al.* (2020) [41]
			79 (79)	N	G		
			187 (186)	G	S		
			205 (204)	Y	H		
			331 (330)	S	F		
			385 (384)	D	N		
			474 (473)	P	S		
		(Go) VP4	168 (169)	K	T		
			343 (342)	V	L		
			385 (384)	D	N		
			471 (470)	S (S)	L (L)		
			666 (665)	F	S		
			677 (676)	D	N		
			713 (712)	D	N		
		(OSU) VP4	145 (145)	K	T		
			178 (178)	D	N		
			185 (185)	I	T		
			267 (265)	Y	C		
			393 (391)	D	H		
			684 (682)	D	H		
		(Wa) VP7	98 (98)	A	V		
			158 (158)	E	K		
			275 (275)	S	P		
			290 (290)	T	I		
		(M) VP7	75 (75)	T (T)	L (P)		
		(Go) VP7	no change				
		(OSU) VP7	no change				
Sequences were determined of Rotarix vaccine-derived isolates from 30 rotavirus-positive infants with gastroenteritis to screen for potential regaining-of-virulence markers. More than half of the nt pointmutations were located on VP4 (60%, 43/72), with a single isolate harboring 5 of them. Fewer mutations were found on NSP4 (14%, 10/72) and VP7 (9%, 6/72); whereas the NSP2, NSP3 and NSP5 did not harbor any changes. Although unique aa substitutions were present, several of them were shared by multiple individuals. In particular, F167L on VP4 was shared by 17 infants (57%), I389L on VP4 by 8 infants (27%), A613V on VP3 by 5 infants (17%), and I45T on NSP4 by 4 infants. These shared NSP4 and VP4 changes are indicated in the right column.	In Belgium, 80 vaccine-derived strains in 5125 rotavirus-positive infants with gastroenteritis from 2007 to 2018. Among the 45 patients with known vaccination status, 39 were vaccinated and 87% received the vaccine less than a month before the gastroenteritis episode. Reconstruction of 30 near complete vaccine-derived genomes revealed 0–11 mutations per genome, with 88% of them being non-synonymous.	NSP4	45 (45)		I (M)	T (T)	Simsek *et al.* (2022) [49]
		VP4	167 (168)		F	L	
			389 (388)		I	L	
VP4 sequence comparison between wild-type (EW) and cell-culture-adapted (ETD_822) murine RV, and using reverse genetics to assess them and their chimera for virulence. Especially an EW VP4 fragment including the differences D452 and S470 compared to RV enhanced virulence.	VP4 sequences were compared bewteen EW (vir.) and ETD_822 (atten., passaging history unknown). RV D6/2 is a natural mono-reassortant strain with cell-culture-adapted simian RRV strain VP4 and all other segments of wild-type murine EW. Using an RV D6/2-based reverse genetic system, VP4 of EW and ETD_822, and their chimeric sequences, were compared for virulence. This concluded virulence at the partial segment (with >1 residue differences) level (at the right, virulent residue sets are indicated by red boxing, with thicker lines for a higher impact).	VP4	80 (80)	Y	H		Kawagishi *et al.* (2024) [18]
			452 (452)	D	N		
			470 (470)	S (S)	L (L)		
			612 (612)	T (T)	A (A)		
			711 (711)	A	T		
Multiple virulent human RV strains were passaged in cel culture, and VP4 sequences were determined. The finding that independent cell-culture passaging of similar or different strains regularly resulted in the acquisition of similar mutations suggest a similar mechanism of adaptation.	50 human rotavirus clinical specimens representing five of the genotypes most frequently associated with severe human disease, including G1P[8], G3P[8], G9P[8], G12P[8], and G2P[4] were passaged, each in triplicate, three to five times in primary monkey kidney cells then ten times in the MA104 monkey kidney cell line. From 13 of the 50 specimens, together 25 rotavirus antigen-positive passaging-defined lineages representing all five genotypes were obtained, which tended to replicate more efficiently in MA104 cells at late versus early passage. Sequence comparison of VP4 found that 34 VP4 variant mutations became dominant in at least at least two of the five genotypes (all indicated here at the right) and that 12 of those became dominant in all genotypes (indicated here with thick boxes).	VP4	19 (19)	Y	H		Carter *et al.* (2024) [55]
			52 (52)	H	Y		
			78 (78)	T/S	I		
			106 (106)	I	V		
			108 (108)	V	I		
			113 (113)	D	N		
			120 (120)	N	T		
			145 (146)	G	S		
			150 (151)	D	E		
			162 (163)	R	K		
			173 (174)	V	I		
			195 (194)	G/N	D		
			199 (198)	T	I		
			252 (251)	D	E		
			268 (267)	R	S		
			281 (280)	I	V		
			295 (294)	Y	F		
			360 (359)	A	V		
			380 (379)	T	A		
			383 (382)	S	R		
			385 (384)	D	H		
			388 (387)	I	L		
			390 (389)	V	L		
			467 (466)	F	L		

			546 (545)	S	N		
			560 (559)	V	I		
			580 (579)	V	I		
			586 (585)	A	T		
			587 (586)	V	I		
			590 (589)	W	L		
			604 (603)	V	L		
			608 (607)	A	S		
			617 (616)	N	K		
			621 (620)	K	R		
			689 (688)	F	V		
			708 (707)	T	A		
			738 (737)	T	I		
**Sequence comparison between wild-type human RV strain AU-1 (fecal) and its low-passage cell culture-adapted variant. The only aa change was VP4-P475L.**	**Whole genome sequence comparison of AU-1-wt (isolated from the stool of an infant) and its derivate AU-1-tc that was passaged 5x in MA104 cells and plaque purified revealed only 1 aa difference. Functional analysis for segment or mutation: not performed.**	**VP4**	**475** (473)	**P**	**L**		**Agbemabiese *et al.* (2024) [56]**
**Sequence comparison of recombinant human/simian triple-reassortant (human RV VP4/VP7/VP6 with genotypes G9-P[6]-I2, other segments simian RV) before and after passaging in cell culture. Whole genome sequencing of the passaged virus revealed a single aa substitution (VP4 E263G) that in both the triple-reassortment and a VP4 mono-reassortment improved growth in cell culture. The mutation appears to reduce the VP5 to VP8 binding in the upright spike.**	**A reverse genetics reassortant, simian RV SA11 backbone with human African RV isolate VP4/VP7/VP6, was passaged 10x in MA-104. Whole genome sequencing revealed only 1 aa difference (VP4 E263G). Introduced into either the triple-reassortant or as mono-reassortment, this mutation enhanced replication in MA104 cells. Virulence *in vivo* was not investigated.**	**VP4**	**263** (262)	**E**	**G**		**Valusenko-Mehrkens *et al.* (2024) [45]**
**The 6 aa amino acid mutations that appeared after serial passaging in Vero cells (Resch *et al*. 2020) were introduced into a virulent human CDC-9 P11 reverse genetics system. Residues VP4 AA331 and AA385 each or in combination were associated with increased replication *in vitro* and reduced virulence in neonatal rats, while the AA364 and AA388 mutations might have additive effects on attenuation.**	**Using a reverse genetics system for strain CDC-9 (G1P[8]) passaged 11 times in MA104 cells and still virulent, the 6 aa mutations hat appeared after serial passaging in Vero cells (Resch *et al*. 2020) were introduced individually and/or as sets (the authors were not succesfull in introducing M364I or I388L individually). Rescue mutants S331F or D385H, were shown to increase replication in a human intestinal cell line (Caco-2), with additive effects of M364I and I388L. Neonatal rats infected with the single S331F or D385H mutants showed reduced viral shedding. The clear impact of the S331F and D385H mutations is expressed by adding red boxes to the above summary of the Resch et al. 2020 study.**	**VP4**	**See above (Resch *et al*. 2020)**				**Bessey *et al.* (2025) [46]**
**Sequence comparison of VP4 in Rotarix (RIX4414, vaccine) derived shed virus with the original vaccine.**	**VP4 sequencing of Rotarix-derived viruses from stool from 8 children living in the Amazon region and comparison to the vaccine reference sequence. Functional analysis for segment or mutation: not performed.**	**VP4**	**167** (168)		**F**	**L**	**Silva *et al.* (2026) [57]**
			**215** (214)		**C**	**S**	
			**263** (262)		**E**	**K**	

Reference

Ward RL, Mason BB, Bernstein DI, Sander DS, Smith VE, Zandle GA, Rappaport RS. Attenuation of a human rotavirus vaccine candidate did not correlate with mutations in the NSP4 protein gene. *J. Virol.* 1997, 71, 6267-6270.
Angel J, Tang B, Feng N, Greenberg HB, Bass D. Studies of the role for NSP4 in the pathogenesis of homologous murine rotavirus diarrhea. *J Infect Dis.* 1998, 177, 455-458.
Chang KO, Kim YJ, Saif LJ. Comparisons of nucleotide and deduced amino acid sequences of NSP4 genes of virulent and attenuated pairs of group A and C rotaviruses. *Virus Genes.* 1999, 18, 229-233.
Oka T, Nakagomi T, Nakagomi O. A lack of consistent amino acid substitutions in NSP4 between rotaviruses derived from diarrheal and asymptomatically-infected kittens. *Microbiol. Immunol.* 2001, 45, 173-177.
Ward RL, Kirkwood CD, Sander DS, Smith VE, Shao M, Bean JA, Sack DA, Bernstein DI. Reductions in cross-neutralizing antibody responses in infants after attenuation of the human rotavirus vaccine candidate 89-12. *J. Infect. Dis*. 2006, 194, 1729-1736.
Tsugawa T, Tatsumi M, Tsutsumi H. Virulence-associated genome mutations of murine rotavirus identified by alternating serial passages in mice and cell cultures. *J. Virol.* 2014, 88, 5543-5558.
Tsugawa T, Tsutsumi H. Genomic changes detected after serial passages in cell culture of virulent human G1P[8] rotaviruses. *Infect Genet Evol.* 2016, 45, 6-10.
Mohanty SK, Donnelly B, Dupree P, Lobeck I, Mowery S, Meller J, McNeal M, Tiao G. A Point Mutation in the Rhesus Rotavirus VP4 Protein Generated through a Rotavirus Reverse Genetics System Attenuates Biliary Atresia in the Murine Model. *J. Virol.* 2017, 91, e00510-17.
Kaneko M, Takanashi S, Thongprachum A, Hanaoka N, Fujimoto T, Nagasawa K, Kimura H, Okitsu S, Mizuguchi M, Ushijima H. Identification of vaccine-derived rotavirus strains in children with acute gastroenteritis in Japan, 2012-2015. *PLoS One*. 2017, 12, e0184067.
Resch TK, Wang Y, Moon S, Jiang B. Serial Passaging of the Human Rotavirus CDC-9 Strain in Cell Culture Leads to Attenuation: Characterization from In vitro and In vivo Studies. *J. Virol.* 2020, 94, e00889-20.
Guo Y, Wentworth DE, Stucker KM, Halpin RA, Lam HC, Marthaler D, Saif LJ, Vlasova AN. Amino acid substitutions in positions 385 and 393 of the hydrophobic region of VP4 may be associated with rotavirus attenuation and cell culture adaptation. *Viruses.* 2020, 12, 408-419.
Simsek C, Bloemen M, Jansen D, Descheemaeker P, Reynders M, Van Ranst M, Matthijnssens J. Rotavirus vaccine-derived cases in Belgium: Evidence for reversion of attenuating mutations and alternative causes of gastroenteritis. *Vaccine.* 2022, 40, 5114-5125.
Kawagishi T, Sánchez-Tacuba L, Feng N, Greenberg HB, Ding S. Reverse Genetics of Murine Rotavirus: A Comparative Analysis of the Wild-Type and Cell-Culture-Adapted Murine Rotavirus VP4 in Replication and Virulence in Neonatal Mice. *Viruses.* 2024, 16, 767-778.
Carter MH, Gribble J, Diller JR, Denison MR, Mirza SA, Chappell JD, Halasa NB, Ogden KM. Human Rotaviruses of Multiple Genotypes Acquire Conserved VP4 Mutations during Serial Passage. *Viruses.* 2024, 16, 978-995.
Agbemabiese CA, Dennis FE, Lartey BL, Damanka SA, Nakagomi T, Nakagomi O, Armah GE. Whole Genome Sequences of the Wildtype AU-1 Rotavirus A Strain: The Prototype of the AU-1-like Genotype Constellation. *Viruses.* 2024, 16, 1529-1634.
Valusenko-Mehrkens R, Schilling-Loeffler K, Johne R, Falkenhagen A. VP4 Mutation Boosts Replication of Recombinant Human/Simian Rotavirus in Cell Culture. *Viruses.* 2024, 16, 565.
Bessey TK, Wang Y, Moon S-S, Sanchez-Tacuba L, Jaïs PH, Greenberg HB, Jiang B. Mutations of two amino acids in VP5 mediate the attenuation of human rotavirus vaccine: evidence from in vitro and in vivo studies. *J. Virol.* 2025, 99, e0106725.
Silva MF, Silva BV, Ramalho E, Pimenta YC, Silva LL, Vieira LS, Xavier MP, Olivares AI, Leite JP, Moraes MT. Examination of In Vivo Mutations in VP4 (VP8*) of the Rotarix® Vaccine from Shedding of Children Living in the Amazon Region. *Viruses.* 2026, 18, 70-89.
